# Factors Associated With Neural Tube Defects Among Newborns Delivered at Debre Berhan Specialized Hospital, North Eastern Ethiopia, 2021. Case-Control Study

**DOI:** 10.3389/fped.2021.795637

**Published:** 2022-02-28

**Authors:** Getaneh Baye Mulu, Bantalem Tilaye Atinafu, Fetene Nigussie Tarekegn, Tigist Demssew Adane, Mesfin Tadese, Abate Dargie Wubetu, Worku Misganaw Kebede

**Affiliations:** ^1^Department of Nursing, College of Health Sciences, Debre Berhan University, Debre Berhan, Ethiopia; ^2^Department of Midwifery, College of Health Sciences, Debre Berhan University, Debre Berhan, Ethiopia; ^3^Department of Pyschiatry, College of Health Sciences, Debre Berhan University, Debre Berhan, Ethiopia

**Keywords:** neural tube defect, newborn, associated factors, Debre Berhan, Ethiopia

## Abstract

**Background:**

Neural tube defects are severe congenital malformations secondary to an abnormal closure of the neural tube between third and fourth weeks of gestational ages. Neural tube defects affect birth outcomes worldwide, with an occurrence of 18.6 per 10,000 live births. In addition, neural tube defects are associated with considerable mortality, morbidity, disability, and socio-economical cost.

**Objective:**

To identify factors associated with neural tube defects among newborns delivered at Debre Berhan Comprehensive Specialized Hospital, 2021.

**Methods:**

Facility-based case-control study design was conducted among 381 (127 cases and 254 controls) newborns delivered from June 2019 to June 2021 at Debre Berhan Specialized Hospital. Consecutive and systematic random sampling techniques were used to select cases and controls, respectively. Data were collected using semi-structured checklists. Finally, data were entered using Epidata version 4.2.1 and analyzed using SPSS version 25. In the bivariable logistic regression model, factors with a *p* < 0.20 were entered into multivariable logistic regressions. Statistical significance was declared at a *p* < 0.05.

**Result:**

In this study, 381 newborns (127 cases and 254 controls) participated with a response rate of 100%. In the logistic regression model, mothers who took medication during pregnancy [AOR 1.83 (95% CI 1.08–3.08)], mothers who did not take a balanced diet during pregnancy [AOR 13.46 (95% CI 7.83–23.13)], and mothers who did not take folic acid before and during the first trimester of pregnancy [AOR 1.71 (95% CI 1.01–2.94)] were significantly associated with neural tube defect.

**Conclusion and Recommendation:**

Mothers who took medication during pregnancy, mothers who did not take balanced diets during pregnancy, and mothers who did not take folic acid during pregnancy were the significant factors of neural tube defects. Health care professionals should focus on maternal safe drug prescription, maternal folate intake, and a balanced diet before and during pregnancy.

## Introduction

Neural tube defects (NTDs), classified as a common group of central nervous system anomalies, comprise a major public health problem (1). NTDs bring about structural defects in the spinal cord, brain, and spine of a developing fetus (2). The etiology of neural tube defects is generally believed to be multifactorial. Genetics and environmental factors have been implicated and shown to be important in their occurrence and recurrence (1).

The neural tube closure usually occurs between the third and fourth weeks of intrauterine life. NTDs are the second most common class of malformations after cardiac anomalies (3). Spinal bifida, encephalocele, and anencephaly are major subgroups of these congenital disabilities (4). Early screening programs are beneficial to decrease mortality and morbidity; furthermore, it allows the families to make informed reproductive choices and design appropriate prenatal care and delivery (5). As a result, in the last previous three decades, the prevalence of NTD has declined in developed countries (5–7).

The incidence of NTDs in 18 low- and middle-income countries of six WHO regions based on live births was 1.67 per 1,000 births for total NTD incidence, 1.13 per 1,000 births for spina bifida, 0.25 per 1,000 for anencephaly, and 0.15 per 1,000 for encephalocele (8). The global estimation indicates that 270,000 neonatal deaths were related to congenital anomalies, while NTDs are one of the most severe and predominant defects as the 2010 WHO report revealed (2, 7). In addition, WHO 2015 report nearly 300,000 babies are born each year with NTDs, resulting in 88,000 newborn deaths and 8.6 million DALYs (3).

The death risks and the severity of the lesion are determined by factors such as the availability of medical and surgical resources (9). NTDs could result in substantial death, morbidity, disability, and other socioeconomic costs (10–12). Different studies conducted in Asia and Africa showed the highest proportion of NTD-associated stillbirths, accounting for 85% of all NTD-related stillbirths occurring worldwide. Furthermore, of the approximated NTD affected live births globally in 2015, about 80% were estimated to die before reaching 5 years (2).

The other retrospective study done in Black Lion tertiary care hospital indicates the most common defect was seen in myelomeningocele (64.4%), followed by meningocele (18.3%) and encephalocele (13.0%). In addition, associated anomalies like clubfoot, undescended testis, different types of hernias, and hydroceles were noted in 28.7% of the patients (13).

Different studies revealed that routine *in utero* early diagnosis and termination of affected pregnancies decrease the prevalence of NTDs. Furthermore, advancement in folic acid provision widely reduces the prevalence of NTDs (7, 14). Globally, NTDs studies identified different factors including low socioeconomic status, maternal exposure to certain environmental factors (i.e., pesticides and chemicals), smoking habit during pregnancy, biological factors, pregnancy in late age, low folic acid supplementation before or during pregnancy, sex of the neonate, and no history of antenatal care (6, 7, 11, 15, 16). A study conducted in Addis Ababa indicates maternal fever, family history of NTDs, drinking coffee, and maternal age <19 and 25–29 were associated with the risk of NTD.

Factors associated with NTD in Ethiopia are not well-investigated, and the country has no national preventive strategies. Some studies recommend case-control and prospective follow-up studies to clearly understand determinants of neural tube defects (17, 18). Thus, the current study aimed to identify the factors associated with NTDs among newborns delivered in Debre Berhan's Comprehensive Specialized Hospital.

## Methods and Materials

### Study Area, Period, and Setting

The study was conducted from July 1, 2021 to August 15, 2021, among newborns delivered at Debre Berhan Specialized Hospital (DBSH). Debre Berhan was one of the earliest capital cities of Ethiopia and the Kingdom of Shoa. This town has an estimated population size of 74,049: 17,221 households; 40,565 were female, 33,483 were male. There are now 10,026 children under the age of 5 years in the town. DBSH is a 200-bed facility with a catchment population of nearly 3 million people (19). More than 350 healthcare providers are employed in the hospital: 40 physicians, 200 nurses, 26 midwives, seven anesthetists, 31 laboratory technicians, two physiotherapists, four dentists, six radiographers, four optometrists, five psychiatrists, and 36 pharmacists (20).

### Study Design

The institutional-based, un-matched case-control study design was conducted among newborns delivered in the Debre Berhan Comprehensive Specialized Hospital, Ethiopia 2021.

### Population

By reviewing maternal medical records, the study population consisted of all newborns delivered at Debre Berhan Comprehensive Specialized Hospital from June 1, 2019 to June 1, 2021. According to the ICD-10 criteria, NTDs cases include anencephaly, spinal bifida, myelomeningocele, and encephalocele occurring either separately or in combination with other congenital malformations (21), whereas the controls are newborn babies without NTDs (22).

### Eligibility Criteria

The study included all mothers who delivered in Debre Berhan Comprehensive Specialized Hospital with complete records of patient's chart, whereas incomplete medical records were excluded.

### Sample Size Calculation and Sampling Technique

The sample size was determined using open Epi version 7.1 using the double population proportion difference formula by considering maternal age (controls exposed 46.1% with AOR 1.96) (6) as independent determinant variables, which gives the maximum sample size compared to other predictor variables. Considering the assumptions (95% CI, 80% power, and 1:2 case to control ratio), an estimated sample size was 345. After adding a 10% non-response rate, the final sample size was 381 (127 cases and 254 controls).

### Sampling Procedure

Based on the pre-study maternal medical records, newborns delivered from June 1, 2019 to June 1, 2021 in Debre Berhan Specialized Hospital were 3,600. From this, 1,400 maternal medical records were incomplete medical records and were dropped out. From the remaining 2,200 maternal medical records, 150 were cases, and 2,050 were controls. Based on the case definition, NTDs include anencephaly, spinal bifida, myelomeningocele, and encephalocele, occurring either separately or in combination with other congenital malformations. The controls are newborn babies born without NTDs in the same hospital from which cases were drawn. Cases are selected by consecutive sampling, whereas two consecutive controls are sampled systematically every eight intervals (2050/254 = 8) from which cases were drawn.

### Variables

#### Dependent Variables

Neural tube defect (**Yes/No**).

#### Independent Variables

Socio-demographic factors: age, sex of the fetus, level of education, income, residence.

Maternal and neonatal: body mass index, comorbid illness, planned pregnancy, ANC follow-up, and previous history of NTD.

Medication-related factors: drug use, folic acid or multivitamin supplement.

Environmental factor: chemical exposure, radiation/heat exposure.

#### Operational Definitions and Key Terms

Neural tube defects are structural defects of the central nervous system that affect the brain, spine, and spinal column throughout embryonic development.

Myelomeningocele is a state where the spinal cord and the tissues covering it protrude out of an opening in the back.

Encephalocele is a type of NTD that causes a sac-like protrusion of the brain and its surrounding membranes through an opening in the skull.

Drugs in this study means both prescribed medications and over the counter.

Periconceptional folic acid supplementation: standard recommendation of folic acid (400 μg/day) for all women 1 month before conception until 12 weeks of gestation should take folic acid supplements (23).

Periconceptional period: a time 1 month before conception until 12 weeks of gestation (23).

Chemical exposure: maternal chemical exposure to aromatic solvents and chlorinated aliphatic solvents (menthanes, ethane, and ethenes) during early pregnancy (24).

Mothers who take enough balanced diet during pregnancy was assessed as intake of at least two additional meals per day during the pregnancy (25).

### Data Collection Tools and Procedures

Trained midwives and nurses collected data from maternal medical records using a checklist. The structured checklist was adapted from a similar study (26–28). The checklist was designed to obtain information on factors associated with neural tube defects for socio-demographic, maternal, and other factors.

### Data Quality Control

To maintain data quality obtained from the maternal medical records, checklists were used. The documentation of information in the checklists was done by midwives and nurses working at the delivery wards. The checklists were primarily prepared in English and translated to Amharic and back to English to check for language consistency by an independent academic translator. Then, experts in related fields evaluated the checklists. The data collection instruments have been pre-tested on 5% of the Mehal Meda General Hospital sample to check language clarity, appropriateness of data collection tools, and estimated time required. Based on the result, necessary amendments were considered. In addition, training has been given regarding the data collection tool and data collection process for both data collectors and supervisors. During the data collection time, close supervision was carried out by supervisors and investigators to ensure data quality. Lastly, data were checked by the supervisor and investigator for completeness and consistency. A random selection of questionnaires has done consistently.

### Data Processing and Analysis

Before beginning analysis, data were cleaned, edited, and coded. Any errors identified at this point were corrected after reviewing the original data using the code numbers. Then, data were entered using Epi-data version 4.2.1 and analyzed by using SPSS 25 statistical software. Frequency distribution and summary statistics such as mean and standard deviation have been calculated for both cases and the controls group. The regression model assumption was also checked to identify and exclude variables that cause poor fit in the model. Bivariable regression models were fitted for each explanatory variable. Factors with a *p*-value less than or equal to 0.20 in the bivariable analysis were fitted to the final multivariable analysis model to control confounders and identify the independent effect of different factors for the occurrence of NTDs. Multicollinearity was checked and fitted by using tolerance and variance inflation factor. Furthermore, in the multivariable logistic regression models, the goodness of fit Hosmer Lemeshow was checked and fitted for the data with the *p*-value of 0.101. Factors having *p* < 0.05 in the multivariable logistic regression model were considered statistically significant. In the end, results were presented in the form of graphs and tables.

## Results

### Socio-Demographic Characteristics of Mothers and Newborns

In this study, a total of 381 newborns (127 cases and 254 controls) were included, which made the response rate 100%. Nearly half of the participants were mothers aged 27–35 in both cases and controls. In addition, half of the newborns were equally females and males in both cases and the control group (Table 1).

**Table 1:** Frequency distribution of socio-demographic characteristics among case and control groups in Debre Berhan Specialized Hospital, 2021.

	**Controls (*****n*** **=** **254)**		**Cases (*****n*** **=** **127)**	
**Variables**	***N***	**(%)**	***N***	**(%)**
**Maternal age**				
18–26	97	38.2	45	35.
27–35	120	47.2	62	49.2
≥36	37	14.6	20	15.75
**Marital status**				
Unmarried	34	13.4	5	3.93
Married	210	82.7	117	92.12
Divorced	10	3.9	5	3.94
**Residence**				
Rural	90	35.4	45	35.4
Urban	164	64.6	82	64.6
**Sex of fetus**				
Male	164	64.6	82	64.5
Female	90	35.4	45	35.5

### Maternal and Obstetric Factors

More than one-third of ANC follow-up visits (35.4%) were recorded among cases and controls group, whereas nearly two-thirds (64.6%) of the cases and controls group have no ANC follow-up visits (Table 2).

**Table 2:** Frequency distribution of maternal and obstetric factors among case and control groups in Debre Berhan Specialized Hospital, 2021.

	**Controls (*****N*** **=** **254)**		**Cases (*****N*** **=** **127)**	
**Variables**	***N***	**%**	***N***	**%**
**ANC follow-up**				
Yes	90	35.4	45	35.4
No	164	64.6	82	64.6
**Mother comorbid illness**			
Yes	19	7.5	18	14.1
No	235	92.5	109	85.9
**History of abortion**			
Yes	120	47.2	60	47.2
No	134	52.8	67	52.8
**History of stillbirth**			
Yes	90	35.4	45	35.4
No	164	64.6	82	64.6
**Status of the current pregnancy**			
Planned	104	40.9	45	35.4
Unplanned	150	59.1	82	64.6
**Number of gestation**				
Singleton	224	88.2	112	88.2
Multiple	30	11.8	15	11.8
**Gestational age**			
<37	30	11.8	15	11.8
37–42	224	88.2	112	88.2
**Mode of delivery**				
SVD	194	76.4	97	76.4
By vacuum	60	23.6	30	23.6
**Mothers who take enough balanced diet**		
Yes	180	70.9	37	29.1
No	74	29.1	90	70.9

*NB, nota bene; ANC, antenatal care; SVD, spontaneous vaginal delivery*.

### Medication-Related Factors

Regarding supplements, nearly one-third of the case group mothers (30.7%) used folic acid (FA) before and during the first trimester of pregnancy, whereas 44.5% control group mothers did not use FA before and during the first trimester of pregnancy (Table 3).

**Table 3:** Frequency distribution of medication-related factors among case and control groups in Debre Berhan Specialized Hospital, 2021.

**Variables**	**Controls (*****n*** **=** **254)**		**Cases (*****n*** **=** **127)**	
	***N***	**(%)**	***N***	**(%)**
**Folic acid supplement**				
Yes	113	44.5	39	30.7
No	141	55.5	88	69.3
**Past medication history**		
Yes	100	39.4	94	74
No	154	60.6	33	26

### Types of Neural Tube Defect

In this study, three-fourth of the cases, 95 (75%), were with encephalin; nearly half of the cases, 61 (48%), were with spina bifida; and the remaining, 5 (4%), were with anencephaly.

### Factors Associated With NTD

Those factors like mothers who took medication during pregnancy, mothers who did not take a balanced diet during pregnancy, mothers who did not take FA before and during pregnancy, comorbid illness during pregnancy, past medication history, balanced diet history, history of stillbirth, folic acid supplements, history of abortion, the status of the current pregnancy, sex of the fetus, maternal ANC follow-up, and residence had a *p* < 0.25 in bivariable logistic regression analysis. Therefore, those factors were entered into a multivariable logistic regression to adjust confounding.

Independent factors having a statistically significant association with neural tube defect in multivariable logistic regressions were mothers taking medication during pregnancy, mothers who did not take FA during pregnancy, and mothers who did not take a balanced diet.

In this study, neural tube defect was associated with mothers who took medication during pregnancy. Mothers who took medication during pregnancy were two times more likely to give birth NTDs than mothers who did not take medication during pregnancy [AOR 1.83 (95% CI 1.08–3.08), *P* = 0.02].

Furthermore, mothers who did not take a balanced diet during pregnancy were 13 times more likely to deliver newborns with NTDs than their counterparts [AOR 13.46 (95% CI 7.83–23.13), *P* = 0.001]. Likewise, the odds of giving birth NTD was nearly two times more likely among mothers who did not take folic acid during pregnancy than mothers who took FA during pregnancy [AOR 1.71 (95% CI 1.01–2.94), *P* = 0.04] (Table 4).

**Table 4:** Bivariable and multivariable results for the factors associated with NTDs among newborns in Debre Berhan Specialized Hospital, 2021.

**Variables**	**Case (*N* = 127)**	**Control (*N* = 254)**	**COR (95% CI)**	**AOR (95% CI)**
	***N* (%)**	***N* (%)**		
**Comorbid illness during pregnancy**				
Yes	18 (14.1)	19 (7.5)	2.04 (1.03–3.4)	1.68 (0.313–3.4)
No	109 (85.9)	235 (92.5)	1	1
**Past medication history**				
Yes	94 (74)	100 (39.4)	4.4 (1.95–6.67)	1.83 (1.08–3.08)^*^
No	33 (26)	154 (60.6)	1	
**A balanced diet during pregnancy**				
Yes	37 (29.1)	180 (70.9)	1	
No	90 (70.9)	74 (29.1)	5.91 (2–8.6)	13.46 (7.83–23.13)^**^
**History of stillbirth**				
Yes	45 (34.5)	90 (35.4)	1 (0.63–1.4)	1.30 (0.96–2.4)
No	82 (65.5)	164 (64.6)	1	1
**Folic acid supplements**				
Yes	39 (30.7)	113 (44.5)	1	1
No	88 (69.3)	141 (55.5)	1.8 (1.15–2.83)	1.71 (1.01–2.94)^*^
**History of abortion**				
Yes	60 (47.2)	120 (47.2)	1 (0.3–2.04)	1.07 (0.99–1.3)
No	67 (52.8)	134 (52.8)	1	1
**Status of the current pregnancy**				
Planed	45 (35.4)	104 (40.9)	1	1
Unplaned	82 (64.6)	150 (59.1)	1.26 (0.96–2.4)	0.924 (0.45–1.89)
**Sex of fetus**				
Male	82 (64.6)	164 (64.6)	1	1
Female	45 (35.4)	90 (35.4)	1 (0.33–2.2)	1.06 (0.42–1.67)
**Maternal ANC follow-up**				
Yes	45 (35.4)	90 (35.4)	1	1
No	82 (64.6)	164 (64.6)	1 (0.47–1.59)	2.05 (1.45–3.3)
**Residence**				
Rural	45 (35.4)	90 (35.4)	1.001 (0.64–1.43)	1.3 (0.136–2.6)
Urban	82 (64.6)	164 (64.6)	1	1

* *Statistically significant with p <0.05*.

** *Statistically significant with p <0.01*.

*** *Statistically significant with p <0.001*.

## Discussion

Neural tube defects remain of public health importance in the cause of morbidity and mortality among newborns and infants. Therefore, this study was conducted on factors associated with neural tube defects among newborns delivered at Debre Berhan Comprehensive Specialized Hospital.

Factors that have a significant association with NTD were mothers who took medication during pregnancy, mothers who did not take FA before and during the first 3 months of pregnancy, and mothers who did not take balanced diets during pregnancy.

In this study, mothers who took medication during pregnancy had nearly two times more likely to have a newborn with a neural tube defect than their counterparts [AOR 1.83 (95% CI 1.08–3.08)]. This finding is consistent with studies conducted in Addis Ababa and the Amhara region (29) and the United States of America (13). This might be because private drug vendors are selling most drugs and shops with and without prescriptions. As a result, mothers are more likely to use medications/over-the-counter medicines during their early pregnancy, resulting in neural tube defects (29–35).

Moreover, mothers who did not take FA supplementation before and during first-trimester pregnancy were nearly two times more likely to deliver newborns with NTDs than mothers who took folic acid before and during pregnancy [AOR 1.71 (95% CI 1.01–2.94)]. This result is similar to a study conducted in Northwestern Ethiopia (36), Addis Ababa (7), Addis Ababa and Amhara region (29), Bale zone (37), and England, Wales (9). This may be because folic acid plays a vital role in synthesizing nucleic acid, lipids, and proteins required for cell division. In addition, iron folate/folic acid plays a role in amino acid metabolisms needed for DNA and RNA synthesis and plays a vital role as an antioxidant agent. Thus, it should be available to all women before and during pregnancy (29).

This study also revealed that NTDs were also predicted by maternal nutritional intake during pregnancy. Mothers who did not take balanced diets during pregnancy were 13.6 times more likely to deliver newborn babies with NTDs than mothers who took a balanced diet [AOR 13.46 (95% CI 7.83–23.13)]. This result is also equivalent to a study conducted in California (38). This may be due to maternal balanced diets like zinc supplements during pregnancy related to essential fetal growth and development. Those supplements also facilitate gene transcription and are needed for cell division, development, and differentiation (39, 40).

## Conclusion and Recommendation

Mothers who took medication during pregnancy, mothers who did not take a balanced diet during pregnancy, and mothers who did not take FA before and during the first trimester of pregnancy were significant predictors of neural tube defects. Therefore, health care professionals should focus on maternal safe drug prescription, maternal folate intake, and a balanced diet before and during pregnancy.

## Data Availability Statement

The original contributions presented in the study are included in the article/supplementary material, further inquiries can be directed to the corresponding author/s.

## Ethics Statement

The studies involving human participants were reviewed and approved by the Institutional Review Board (5) of the College of Medicine and Health Sciences, Debre Berhan University, with IRB Protocol number 059/21/CHS. Further, a permission letter was taken from Debre Berhan comprehensive specialized Hospital administration to utilize the birth records and submitted to the documentation center. The study was conducted based on the STROBE guideline for a case-control study. Written informed consent for participation was not required for this study in accordance with the national legislation and the institutional requirements.

## Author Contributions

GM, BA, FT, and TA conceived the study, performed the data analysis, contributed to data interpretation and drafting, and critically reviewed the manuscript. MT, AW, and WK designed the study, performed the data analysis, contributed to data interpretation, and critically reviewed the manuscript. In addition, GM, BA, FT, TA, MT, AW, and WK contributed to the performance of data, drafted the manuscript, and critically reviewed the manuscript. All authors read and approved the final manuscript.

## Conflict of Interest

The authors declare that the research was conducted in the absence of any commercial or financial relationships that could be construed as a potential conflict of interest.

## Publisher's Note

All claims expressed in this article are solely those of the authors and do not necessarily represent those of their affiliated organizations, or those of the publisher, the editors and the reviewers. Any product that may be evaluated in this article, or claim that may be made by its manufacturer, is not guaranteed or endorsed by the publisher.

## Abbreviations

AEDs: Anti-epileptic drugs
ANC: Ante Natal Care
APH: Anti PartumHemorrhage
BD: Birth Defect
CS: Cesarean Section
DALYS: Disability-Adjusted Life Years
EDHS: Ethiopian Demographic and Health Survey
FHB: Fetal Heart Beat
END: Early Neonatal Death
GA: Gestational Age
IHRERC: Institutional Health Research Ethics Review Committee
LBs: Live Births
NICU: Neonatal Intensive Care Unit
NTDs: Neural Tube Defect
UK: United Kingdom
WHO: World Health Organization
FA: Folic Acid.

## References

[1] Christianson AL Howson CP Modell B. Global Report on Birth Defects: The Hidden Toll of Dying and Disabled Children. March of Dimes Birth Defects Foundation (2006).

[2] Taruscio D. Folic Acid: From Research to Public Health Practice. Istituto Superiore di Sanità (2004).

[3] Zaganjor I Sekkarie A Tsang BL Williams J Razzaghi H Mulinare J. Describing the prevalence of neural tube defects worldwide: a systematic literature review. PLoS ONE. (2016) 11:e0151586. 10.1371/journal.pone.0151586 27064786 PMC4827875

[4] Hassan AM. Prevalence, associated factors and outcome of neural tube defects: a retrospective study. Biomed Pharmacol J. (2021) 14:2. 10.13005/bpj/2175

[5] Williams J Mai CT Mulinare J Isenburg J Flood TJ Ethen M. Updated estimates of neural tube defects prevented by mandatory folic acid fortification-United States, 1995-2011. MMWR Morb Mortal Weekly Rep. (2015) 64:1. 25590678 PMC4584791

[6] Berihu BA Welderufael AL Berhe Y Magana T Mulugeta A Asfaw S. Maternal risk factors associated with neural tube defects in Tigray regional state of Ethiopia. Brain Dev. (2019) 41:11–8. 10.1016/j.braindev.2018.07.013 30075882

[7] Gedefaw A Teklu S Tadesse BT. Magnitude of neural tube defects and associated risk factors at three teaching hospitals in Addis Ababa, Ethiopia. Biomed Res Int. (2018) 2018:4829023. 10.1155/2018/4829023 29713643 PMC5866884

[8] Lo A Polšek D Sidhu S. Estimating the burden of neural tube defects in low-and middle-income countries. J Global Health. (2014) 4:1. 10.7189/jogh.04.010402 24976961 PMC4073251

[9] Morris J Wald N. Quantifying the decline in the birth prevalence of neural tube defects in England and Wales. J Med Screen. (1999) 6:182–5. 10.1136/jms.6.4.182 10693061

[10] Christianson A Howson CP Modell B. March of Dimes: Global Report on Birth Defects, The Hidden Toll of Dying and Disabled Children (2005).

[11] Behrooz A Gorjizadeh MH. Prevalence and correlates of neural tube defect in south West Iran: retrospective analysis. Sultan Qaboos Univers Med J. (2007) 7:31. 21654942 PMC3086415

[12] Greene ND Copp AJ. Neural tube defects. Annu Rev Neurosci. (2014) 37:221–42. 10.1146/annurev-neuro-062012-170354 25032496 PMC4486472

[13] Taye K Bedru A. Pattern of neural tube defects at Tikur Anbessa Hospital, Addis Ababa, Ethiopia. Ethiopian Med J. (2009) 47:71–6. 19743784

[14] Muller P Wilkinson C Cocciolone R Haan E Chan A. Trends in state/population-based screening for neural tube defects (NTDS), south australia 1986-2004. Am J Obstetr Gynecol. (2006) 195:S194. 10.1016/j.ajog.2006.10.698

[15] World Health Organization. Congenital Anomalies 2016 (2016).

[16] Øyen N Olsen SF Basit S Leirgul E Strøm M Carstensen L. Association between maternal folic acid supplementation and congenital heart defects in offspring in birth cohorts from Denmark and Norway. J Am Heart Assoc. (2019) 8:e011615. 10.1161/JAHA.118.011615 30857459 PMC6475034

[17] Sorri G Mesfin E. Patterns of neural tube defects at two teaching hospitals in Addis Ababa, Ethiopia a three years retrospective study. Ethiopian Med J. (2015) 53:119–26. 26677521

[18] CSA-Ethiopia I. International: Ethiopia Demographic and Health Survey 2011. Central Statistical Agency Addis Ababa, Ethiopia ICF International. Calverton, MD (2012).

[19] Sharew NT Bizuneh HT Assefa HK Habtewold TD. Investigating admitted patients' satisfaction with nursing care at Debre Berhan Referral Hospital in Ethiopia: a cross-sectional study. BMJ Open. (2018) 8:e021107. 10.1136/bmjopen-2017-021107 29773703 PMC5961596

[20] Kolola T Gezahegn T. A twenty-four-hour observational study of hand hygiene compliance among healthcare workers in Debre Berhan referral hospital, Ethiopia. Antimicrob Resist Infect Control. (2017) 6:1–5. 10.1186/s13756-017-0268-y 29093813 PMC5663127

[21] World Health Organization. The International Statistical Classification of Diseases and Health Related Problems ICD-10: Tenth Revision (Geneva) (2004).

[22] Gashaw A Shine S Yimer O Wodaje M. Risk factors associated to neural tube defects among mothers who gave birth in North Shoa Zone Hospitals, Amhara Region, Ethiopia 2020: Case control study. PLoS ONE. (2021) 16:e0250719. 10.1371/journal.pone.0250719 33901231 PMC8075213

[23] Tesfay FA Aga FB Teshome GS. Determinants of neural tube defect among children at zewditu memorial hospital, addis ababa, ethiopia a case control study. Int J Afr Nurs Sci. (2021) 2021:100318. 10.1016/j.ijans.2021.100318

[24] Desrosiers TA Lawson CC Meyer RE Richardson DB Daniels JL Waters MA. Maternal occupational exposure to organic solvents during early pregnancy and risks of neural tube defects and orofacial clefts. Occup Environ Med. (2012) 69:493–9. 10.1136/oemed-2011-100245 22447643 PMC3719396

[25] Ngwira A Stanley CC. Determinants of low birth weight in Malawi: Bayesian geo-additive modelling. PLoS ONE. (2015) 10:e0130057. 10.1371/journal.pone.0130057 26114866 PMC4482619

[26] Makelarski JA. Selected Enviromental Exposures and Risk of Neural Tube Defects. The University of Iowa (2010).

[27] Salih MA Murshid WR Mohamed AG Ignacio LC de Jesus JE Baabbad R. Risk factors for neural tube defects in Riyadh City, Saudi Arabia: Case-control study. Sudanese J Paediatr. (2014) 14:49. 27493405 PMC4949798

[28] Wang M Wang Z-P Gao L-J Yang H Zhao Z-T. Maternal consumption of non-staple food in the first trimester and risk of neural tube defects in offspring. Nutrients. (2015) 7:3067–77. 10.3390/nu7053067 25919306 PMC4446739

[29] Taye M Afework M Fantaye W Diro E Worku A. Factors associated with congenital anomalies in Addis Ababa and the Amhara Region, Ethiopia: a case-control study. BMC Pediatr. (2018) 18:142. 10.1186/s12887-018-1096-9 29699508 PMC5921791

[30] van Gelder MM Bos JH Roeleveld N de Jong-van den Berg LT. Drugs associated with teratogenic mechanisms. Part I: dispensing rates among pregnant women in the Netherlands, 1998-2009. Human Reproduct. (2014) 29:161–7. 10.1093/humrep/det369 24105826

[31] Van Gelder MM Van Rooij IA Miller RK Zielhuis GA de Jong-van den Berg LT Roeleveld N. Teratogenic mechanisms of medical drugs. Human Reproduct. (2010) 16:378–94. 10.1093/humupd/dmp052 20061329

[32] Mohammed MA Ahmed JH Bushra AW Aljadhey HS. Medications use among pregnant women in Ethiopia: a cross sectional study. J Appl Pharmaceut Sci. (2013) 3:116. 10.7324/JAPS.2013.3421

[33] Admasie C Wasie B Abeje G. Determinants of prescribed drug use among pregnant women in Bahir Dar city administration, Northwest Ethiopia: a cross sectional study. BMC Pregnancy Childbirth. (2014) 14:325. 10.1186/1471-2393-14-325 25233893 PMC4177766

[34] Kebede B Gedif T Getachew A. Assessment of drug use among pregnant women in Addis Ababa, Ethiopia. Pharmacoepidemiol Drug Saf. (2009) 18:462–8. 10.1002/pds.1732 19334033

[35] Abeje G Admasie C Wasie B. Factors associated with self medication practice among pregnant mothers attending antenatal care at governmental health centers in Bahir Dar city administration, Northwest Ethiopia, a cross sectional study. Pan Afr Med J. (2015) 20:276. 10.11604/pamj.2015.20.276.4243 26161199 PMC4483357

[36] Seyoum G Adane F. Prevalence and associated factors of birth defects among newborns at referral hospitals in Northwest Ethiopia. Ethiopian J Health Dev. (2018) 32:1–7.

[37] Atlaw D Worku A Taye M Woldeyehonis D Muche A. Neural tube defect and associated factors in Bale zone hospitals, southeast Ethiopia. J Pregnancy Child Health. (2019) 6:412. 10.4172/2376-127X.1000412

[38] Velie EM Block G Shaw GM Samuels SJ Schaffer DM Kulldorff M. Maternal supplemental and dietary zinc intake and the occurrence of neural tube defects in California. Am J Epidemiol. (1999) 150:605–16. 10.1093/oxfordjournals.aje.a010059 10490000

[39] Vallee BL Falchuk KH. The biochemical basis of zinc physiology. Physiol Rev. (1993) 73:79–118. 10.1152/physrev.1993.73.1.79 8419966

[40] Tamura T Goldenberg RL. Zinc nutriture and pregnancy outcome. Nutr Res. (1996) 16:139–81. 10.1016/0271-5317(95)02068-3

